# Making myself understood: perceived factors affecting the intelligibility of sung text

**DOI:** 10.3389/fpsyg.2014.00809

**Published:** 2014-09-04

**Authors:** Philip A. Fine, Jane Ginsborg

**Affiliations:** ^1^Department of Psychology, University of BuckinghamBuckingham, UK; ^2^Centre for Music Performance Research, Royal Northern College of MusicManchester, UK

**Keywords:** singing, sung text, intelligibility, understanding, performer, lyrics

## Abstract

Singing is universal, and understanding sung words is thought to be important for many listeners’ enjoyment of vocal and choral music. However, this is not a trivial task, and sung text intelligibility is probably affected by many factors. A survey of musicians was undertaken to identify the factors believed to have most impact on intelligibility, and to assess the importance of understanding sung words in familiar and unfamiliar languages. A total of 143 professional and amateur musicians, including singers, singing teachers, and regular listeners to vocal music, provided 394 statements yielding 851 references to one or more of 43 discrete factors in four categories: performer-related, listener-related, environment-related and words/music-related. The factors mentioned most frequently in each of the four categories were, respectively: diction; hearing ability; acoustic; and genre. In more than a third of references, the extent to which sung text is intelligible was attributed to the performer. Over 60% of respondents rated the ability to understand words in familiar languages as “very important,” but only 17% when the text was in an unfamiliar language. Professional musicians (47% of the sample) rated the importance of understanding in both familiar and unfamiliar languages significantly higher than amateurs but listed fewer factors overall and fewer listener-related factors. The more important the respondents rated understanding, the more performer-related and environment-related factors they tended to list. There were no significant differences between the responses of those who teach singing and those who do not. Enhancing sung text intelligibility is thus perceived to be within the singer’s control, at least to some extent, but there are also many factors outside their control. Empirical research is needed to explore some of these factors in greater depth, and has the potential to inform pedagogy for singers, composers, and choral directors.

## INTRODUCTION

Singing is universal throughout human society, and in evolutionary terms it may be the oldest type of music, possibly – in a wordless form – even predating language (Mithen, 2005). As a medium of communication, music has many similarities to and differences from language (e.g., Meyer, 1956; Sloboda, 2005; Patel, 2010; Slevc, 2012), but the communication of sung text, when words are combined with melody, clearly has more in common with language than does mere musical expression. It could be argued that one of the singer’s foremost responsibilities is communication, whereby listeners gain some level of understanding of the message being transmitted. In a language familiar to the listener, this involves understanding the individual words. If communication is important, then the intelligibility of those words is even more important. We are here defining intelligibility as the extent to which the speaker’s or singer’s message can be understood by the listener (Munro and Derwing, 1995; Kennedy and Trofimovich, 2008). This can be distinguished from comprehensibility, that is, subjective ease of understanding (Kennedy and Trofimovich, 2008). Indeed, being able to understand the sung text may well contribute greatly to listeners’ enjoyment of vocal music. Collister and Huron (2008) state that the results of their study on sung text intelligibility “…affirm the common experience reported by concertgoers and music listeners that sung lyrics are often unintelligible” (p. 120). Even when listeners are largely unfamiliar with the language being sung, grasping the text could in principle add to their understanding of the overall meaning of the song, perhaps through the identification of one or two familiar words. In certain circumstances, however, listeners may not be interested in understanding the text being sung. Thus the need for sung text to be intelligible depends on the listener’s reason for listening and also on the genre of the music. There may be less need for text intelligibility in the context of background music than when the listener is actively attending to live performance; there may be more need, arguably, in folk songs and opera than in some pop music. Indeed, sung text intelligibility has been shown to be dependent on genre: Condit-Schultz and Huron (2013) suggest that jazz is the most intelligible and classical the least, although they did not differentiate classical music into opera and other sub-genres.

Speech is one of the primary modes of human communication, but its perception is complex. Speech is fast (Liberman et al., 1967), the acoustic signal in speech is almost continuous, and its processing creates challenges for the listener (Harley, 2014). Such challenges include (1) the speed of speech, typically 10 phonemes per second, (2) co-articulation, whereby the acoustic properties of individual speech sounds differ depending on the articulation of the speech sounds on either side, and (3) non-invariance, the fact that the same word can sound different when uttered by different speakers. Cognitive psychologists have investigated how people segment and understand the speech signal (e.g., Butterfield and Cutler, 1988; Casserly and Pisoni, 2010; Samuel, 2011). Context has also been shown to be important in understanding speech: Lieberman (1963) found that many fewer individual words spliced from sentences were understood than when heard in the original sentence context. It is probably safe to assume that many of the same cognitive mechanisms are involved when we listen to sung text, but the fact that sung text involves music introduces additional variables that may interfere with listeners’ comprehension.

Singers are different from instrumentalists in that one of the most important aspects of their music making is the communication of words; they are “expected to keep the musical line flowing appropriately while keeping the text intelligible, a difficult task for anyone” (Dunn, 1997, p. 101). Indeed the potential trade-off between the articulation of consonants, choice of vowels and beauty of sound is perceived as a perennial problem for opera singers: their attempts to perform with clear diction can “severely compromise the musicality of the message” (Scotto Di Carlo, 2007a, p. 564), although we have identified no empirical evidence to support these views. Given Collister and Huron’s (2008) observation that sung lyrics are often unintelligible, producing understandable sung text is certainly likely to be a central concern for singers and, for example, choral conductors. Singers’ training involves the development of the voice as an instrument. The purpose of vocal training is to ensure power and smoothness of tone throughout the whole range of the voice, in terms of both dynamics and pitch. Vocal pedagogy also emphasizes the development of clear, but not over-enunciated, diction (solo singers: Falkner, 1983; Adams, 1999; choral singers: Emmons and Chase, 2006). Clear diction would be expected to lead to more understandable words, and hence a better all-round performance, all other things – such as intonation, for example – being equal. According to Novák and Vokrál (2000, p. 154), “One of the criteria, that is evaluated as a part of singer’s performance, is the intelligibility of the singed text” (*sic*).

When considering how listeners understand spoken text, it is important to consider the characteristics not just of the speaker but also those of the listener, such as familiarity with the language and interest in the topic being communicated, as well as age (Humes, 1996) and the presence of any hearing loss (Humes, 1991). Furthermore, environmental factors, such as room acoustics (e.g., Rychtáriková et al., 2011) and background noise levels, may affect the intelligibility of spoken text. We can assume that, in general, these broad classes of factor (speaker/performer, listener, and environment) apply also to the understanding of sung text, in addition to those factors that relate specifically to the music and how it is used to set the words. Thus intelligibility is likely to be influenced by more than just the singer’s diction. Relating to these other classes of factor, Scotto Di Carlo (2007a) mentions, for instance, the masking effect of orchestral accompaniment, the acoustics of the concert hall and the listeners’ auditory abilities.

Much of the existing research into performer-related factors affecting sung text intelligibility (Scotto Di Carlo, 2007a,b) has focused on the intelligibility of individual vowels and consonant-vowel syllables as a function of pitch (e.g., Sundberg, 1987; Sundberg and Ternström, 2008) and the effect of the singer’s formant (Smith and Scott, 1980; Benolken and Swanson, 1990; Hollien et al., 2000; Gregg and Scherer, 2006; Sundberg and Romedahl, 2009): the cluster of powerful formants around 3 kHz that singers’ training aims to develop, and which aids vocal projection. The findings of this research show that, at higher pitches, particularly in the soprano register, sung vowels become increasingly difficult to distinguish from one another. This has been explained in terms of the relationship between the formant frequencies of the vowels and the resonant frequencies of the vocal tract. Dunn (1997, p. 101) for example, observes that “in the case of performing the notes at upper limits of a singer’s range, certain vowels, and consonants are extremely hard to sing in tune and intelligibly, necessitating the modification of some vowels and some loss of text clarity.” The deliberate modification of vowels in the interests of preserving the musical line (Hollien et al., 2000), consonant confusions (Collister and Huron, 2008) and modifications believed to improve intelligibility (Ginsborg, 2014), and the use of vibrato (Sundberg, 1995) also affect listeners’ understanding of sung text. All these might be thought of as distorting the “natural” attributes of the lyrics. However, there is considerably more to understanding text, whether sung or spoken, than identifying individual vowels and syllables out of context. Recently, researchers have begun to investigate sung text intelligibility for more ecologically valid stimuli and situations. Collister and Huron (2008) and Johnson et al. (2014) have compared the intelligibility of spoken and sung words. These words, too, lacked semantic context, however, as all the utterances to be identified by listeners were of the form “I am singing/saying the word ____.” Edward Wickham and The Clerks, an *a cappella* vocal group that specializes primarily in Renaissance music, are also investigating the effects of certain specific factors on sung text intelligibility, from the standpoint of auditory streaming, using their audiences as participants (Heinrich et al., 2012).

In more ecologically valid musical situations, singers are usually accompanied by other singers and/or instrumentalists. In opera at least (Scotto Di Carlo, 2007a), the balance between the pit orchestra and the on-stage singers is a “determining factor in the intelligibility of the singing voice” (p. 562). The singer’s formant, mentioned above, enables male opera singers, and possibly female singers (although the question as to whether it exists in sopranos is controversial: see for instance Weiss et al., 2001; van der Linde, 2013), to project their voices over orchestras, even those of Wagnerian proportions and power (Sundberg and Romedahl, 2009), and is developed in the course of training. Evidence for the singer’s formant derives from research using participants who sing Western opera, as it is not found in genres such as Chinese opera (Sundberg et al., 2012), musical theater (Björkner, 2008), country singing (Cleveland et al., 2001) and pop singing (Borch and Sundberg, 2002). However, in terms of intelligibility, Sundberg and Romedahl (2009) found that opera singers were less intelligible when singing nonsense syllables than musical theater singers, suggesting that the presence of the singer’s formant may in fact decrease sung text intelligibility. The balance between singer and orchestra is, however, under the control of the singer only to a certain extent. As suggested above, singers undertake vocal training to develop their power and volume, and development of the singer’s formant and the ability to project their voices above the orchestra is a by-product of this training; in addition they learn to vary the volume at which they sing. Nevertheless, the size and intensity of the instrumental or orchestral accompaniment, which also affect balance, are typically beyond the singer’s direct control. In chamber ensembles or other groups of musicians all the performers must take responsibility for balance; in directed ensembles such as orchestras the conductor is in charge. Extra-performer issues relevant to balance include the forces the composer requires and even room or building acoustics. One pertinent example is the underscoring of dialog, as well as songs, in many musical theater productions, such that even if the band are playing as quietly as possible, the audience may still have difficulty understanding what is being said or sung. Parati et al. (2004), Sato et al. (2005), and Sato and Prodi (2009) have investigated the subjective evaluation of the balance between opera singers and the pit orchestra, but to our knowledge the effect of this balance on sung text intelligibility has not been investigated.

As Scotto Di Carlo (2007a) points out, however, the singer can only do so much to ensure intelligibility (Fine and Ginsborg, 2007); the perception of sung text depends to a certain extent on the listener. Factors affecting the listener are also likely to impact on their ability to understand what is being sung. Hearing problems will affect this ability, particularly in the presence of background sounds (Lorenzi et al., 2006), and these are greater for older listeners affected by age-related hearing loss (Humes, 1996). Thus any detrimental effects of accompaniment and subsequent balance problems are likely to be exacerbated by hearing loss and aging. Even if the performer’s diction is clear and the listener’s hearing is normal, it would not be surprising for the level of familiarity with the language being sung, the specific song and even the specific text to affect understanding. Gass and Varonis (1984), for instance, have shown that familiarity with specific non-native speakers or accents, as well as familiarity with the topic, can assist speech comprehension. Furthermore, it is well known that speech perception is easier when the listener is attending actively, as shown, for instance, in studies demonstrating the well-known and much-researched cocktail party effect (Cherry, 1953; Bronkhorst, 2000). We would expect that sung text would similarly be easier to understand when the listener is actively attending to the singer.

This leads us on to the consideration of factors pertaining to the environment. The level of attention paid to the singer is under the listener’s control. This is not, however, the case for external environmental distractions. Any distraction in the environment, whether caused by other audience members, background noise, or even the task the listener is doing, could in principle affect the listener’s ability to understand the sung text. The effect of background noise on speech perception has been widely researched, whether relating to hearing aids and cochlear implants (Nabelek et al., 2004; Gifford and Revit, 2010), the perception of speech in a non-native language (Shimizu et al., 2002), children with language difficulties (Vance and Martindale, 2012) or adults with visual problems (Erber et al., 2000). In all cases, background noise impaired speech comprehension. Background noise would be expected to have a similar effect on the perception of sung text. As mentioned earlier, sung text, unlike speech, is often accompanied by other performers, and thus there is usually some background sound potentially masking the words being sung. This relates to the issue of balance, already discussed as a performer-related factor. We are now considering the environmental balance, however: in other words the signal to noise ratio, where the sung text is the signal, and everything else, both musical and extra-musical, is the noise. So the level of background noise is also an important determinant of sung text intelligibility, as louder background noise is more likely to mask the words.

The acoustics of the room or performance space can likewise affect both spoken and sung text intelligibility (Bradley et al., 1999; Boothroyd, 2004; Scotto Di Carlo, 2007a; Yang and Bradley, 2009; Rychtáriková et al., 2011), due mainly to the filtering and echoing effects of reverberations on the sound signal. A great deal of care is taken when designing bespoke concert halls (Gade, 1990; Beranek, 2004) to create the desired acoustic, but of course many musical performances take place in churches, auditoria, and smaller rooms not specifically designed for music. Many large churches and cathedrals are renowned for their long echoes, and however aesthetically pleasing such an acoustic is for choral music, it certainly does not assist the listener to understand the words.

Some musical performances take place outside, where there may be many fewer surfaces creating reverberations and echoes than indoors, thus producing a very different acoustic. There has been little research on the acoustics of open-air auditoria, other than Chourmouziadou and Kang’s (2008) study of the effects of scenery on the acoustics of ancient theaters, and Barkas’s (2008) study of the acoustic of a city-center open-air theater in Greece, but neither considered its effects on spoken or sung text intelligibility. In such cases the location of the performer in relation to the listener is important, as is any natural acoustic from the landscape, buildings, and so forth. Proximity to the performers is also likely to make them easier to see. It has long been known that seeing the speaker’s mouth can affect speech comprehension, usually improving it (Sumby and Pollack, 1954; Samuel, 2011), as in the case of lip-reading for the hard of hearing, and in artificial situations altering it, as in the McGurk effect (McGurk and MacDonald, 1976). It would be similarly expected that seeing the singer’s lips would help the listener to understand the sung text, and this has recently been demonstrated by Jesse and Massaro (2010).

A further environmental factor likely to affect sung text intelligibility is the use (or abuse) of amplification. This can certainly amplify the singer and address issues of balance, but there is the danger that the sound may be distorted, making intelligibility worse rather than better. The use of amplification also depends very much on genre: microphones are regularly used in pop and musical theater, but to a lesser extent in opera and classical song, for example. Most of the research into the effects of amplification has either investigated the problem of hearing loss caused by too much listening to amplified music (West and Evans, 1990) or the effects of amplification on the quality of sound as experienced by wearers of hearing aids, using measures such as ratings of pleasantness (Davies-Venn et al., 2007) and speech intelligibility (Ching and Dillon, 2013). One study shows that the maintenance of intelligibility when the sound source is subject to high levels of amplification depends on various factors, including room acoustics (Doria et al., 2012). No one seems to have investigated the effects of amplification, however, on the intelligibility of sung words to people with normal hearing.

The main difference between speech and sung text is the presence of the music and the way the words are set to music by the composer. Some composers deliberately make use of natural speech rhythms and intonations in their vocal music – Janáček’s operas are an example of this – but this is comparatively rare. Factors related to word-setting must, therefore, affect listeners’ understanding of the sung text. The use of melisma (a single syllable set to several different notes so that it lasts an unconscionably long time, often heard in the music of Handel and Britten), for example, can affect the listener’s parsing of the words. Although melismatic singing can render sung text less intelligible than syllabic singing (Johnson et al., 2014), it may make sense for composers to write melismatically rather than syllabically at higher registers, owing to the difficulties, outlined earlier, of producing identifiable vowels at high pitch (Scotto Di Carlo, 2007a). Indeed, Scotto Di Carlo (2007a) bemoans the fact that “librettists and composers have not always known how to adapt their text to the capabilities of the human voice” (p. 562). Johnson et al. (2014) also found that sung diphthongs are more intelligible than monophthongs, and archaic forms of language are less intelligible than everyday language. Thus the actual choice of words that the librettist and composer make can affect the potential intelligibility of the text when sung. Finally, some choral music compositions involve polytextuality: multiple texts being sung simultaneously by different singers (Leach, 2010). It is often the case that any words being sung simultaneously with others have already been sung in the absence of other competing words so that the listener has already heard them, or they may already be expected to be familiar to the listeners, for instance from liturgical mass settings.

Much of the research cited above has measured the effects of manipulating various factors on the perception of spoken or sung text. To our knowledge, however, musicians’ perceptions of the factors that influence sung text intelligibility in a wider context have not been investigated. Given this gap in the literature, it is possible that the insights of musicians may shed light on factors hitherto unconsidered. Hence, the rationale for undertaking the present study was to address this by collecting musicians’ perceptions of factors underlying listeners’ understanding of the words of songs and arias in live and recorded performances. So as to reach a wide range of experienced listeners and performers, we therefore carried out an exploratory survey with the participation of musicians who were primarily singers and singing teachers. We also aimed to verify the assumption that sung text intelligibility is important for musicians. The main objectives of the study were to (1) ascertain the importance of understanding sung text to respondents, in both familiar and unfamiliar languages, (2) identify the factors the respondents believe to be the most important influences on intelligibility, through the use of content analysis, and (3) to assess their relative importance, taking into account respondents’ musical expertise. As the study is exploratory, no specific hypotheses were tested.

## MATERIALS AND METHODS

### RESPONDENTS

There were 143 respondents, three of whom did not disclose their sex, aged between 18 and 67 (mean = 35.8, SD = 13.7 years). A total of 85 (61%) were female and 55 (39%) were male. More than three-quarters of the respondents were from the UK (76%); the remainder were from Argentina, Australia, Austria, Canada, Denmark, Finland, France, Germany, Ireland, Poland, and the USA. All but eight of the respondents identified their level of singing experience. For the purposes of analysis, those 76 respondents who described themselves as professional musicians (including accompanists, coaches, conductors, and repetiteurs as well as professional singers), semi-professional singers and student singers were deemed experts (56%) and those 59 respondents who described themselves as amateur singers, occasional singers, and non-singers were deemed non-experts (44%). Eighteen of the experts were or had been singing teachers with a mean of 10.2 years’ teaching experience (SD = 11.0); eight had between 14 and 35 years’ teaching experience. The respondents thus formed a relatively heterogeneous group: this provided a welcome opportunity, since the study was exploratory, to survey a wide range of musician.

### SURVEY

A questionnaire survey was devised, based loosely on that of Himonides and Welch (2006), to address the research objectives outlined above. The questionnaire forms Appendix A. Once ethical approval had been granted by the Research Ethics Committees of both authors’ institutions, respondents’ informed consent was obtained at the start of the questionnaire, followed by demographic data, including sex, age, nationality, profession, and listening preferences. Respondents were then asked to rate on a 3-point scale (not at all important, quite important, very important) how important they felt it was to be able to understand sung text, in both familiar and unfamiliar languages, and the proportions of music they listened to that were vocal and/or choral, as opposed to purely instrumental. Finally, they were asked to provide a list of all the factors they considered important for the intelligibility of sung text, in an open-ended format (examples can be found in the Discussion), under the following five headings: performer-related, listener-related, environment-related, music-related (including the setting of words to music), and other; they were also invited to make further comments both specific to the questions asked and the research in general. One example for each of the first four headings was provided, respectively: diction; hearing ability; location: indoors/outdoors; genre. We bore this in mind when considering the frequency with which specific factors were mentioned. As can be seen in the Results section, all the examples given above, except location, were, perhaps unsurprisingly, those noted most often.

### PROCEDURE

The questionnaire was made available both on the internet (through SurveyMonkey®) and on paper. Respondents were recruited initially via choral societies, singers, and singing teachers known to the researchers (both of whom are expert singers with considerable experience of choral singing) and, subsequently, publicized on the PsyMus email distribution list (see www.sempre.org.uk) and, using “snowball” methods, by word-of-mouth.

### ANALYSIS

The open-ended responses to the questionnaire were analyzed using content analysis (Berelson, 1952; Hsieh and Shannon, 2005). The respondents’ statements were split into sub-statements, each containing an individual word or phrase. These sub-statements were grouped into categories, each sub-statement being a member of only one category. Content analysis allows for quantitative as well as qualitative analysis, and thus the number of instances in each category was considered. As part of this quantitative analysis, we compared various groups of respondents based upon their demographic and rating data, as follows. The respondents were split into subgroups on the basis of (1) level of musical expertise, (2) rating of perceived understanding of sung text in both familiar and unfamiliar languages, (3) proportion of vocal and/or choral music listened to, and (4) whether or not respondents reported being or having been a singing teacher.

## RESULTS

### IMPORTANCE OF UNDERSTANDING TEXT IN FAMILIAR AND UNFAMILIAR LANGUAGES

Overall, the respondents reported listening to a wide range of music genres, the most popular of which was “classical,” though a few respondents listened to whatever their favorite radio station was playing. A total of 132 respondents reported the percentage of their time spent on listening to music that was devoted to vocal and/or choral as opposed to instrumental music. Of these, 67 (51%) reported spending at least 75% of their music-listening time to vocal and/or choral music. As shown in **Table 1**, 107 respondents expressed views as to the importance of understanding sung text in familiar and unfamiliar languages (in addition, one respondent commented only on texts in familiar languages). It was “very important” to 61% when listening to sung text in a familiar language, but only to 17% in an unfamiliar language.

**Table 1:** Numbers and percentages of respondents indicating different levels of importance for understanding texts in familiar and unfamiliar languages.

	Very important	Quite important	Not important
Familiar language (*n*= 108)	66 (61.1%)	35 (32.4%)	7 (6.5%)
Unfamiliar language (*n*= 107)	18 (16.8%)	55 (51.4%)	34 (31.8%)

We coded importance ratings on a scale of 1–3, with 1 = not important and 3 = very important. While respondents rated understanding texts in familiar languages (*M* = 2.54) as significantly more important than understanding them in unfamiliar languages (*M* = 1.85, *t*_106_ = 11.001, *p* < 0.001), there was a strong positive correlation between the importance of understanding texts in familiar and unfamiliar languages (*r_S_* = 0.502, *p* < 0.001). Although respondents differed in the importance they ascribed to intelligibility overall, those who thought it important to understand text in a familiar language still preferred to be able to hear the words even in an unfamiliar language.

### FACTORS BELIEVED TO UNDERLIE THE INTELLIGIBILITY OF SUNG TEXT

Ninety-four of the respondents provided 394 open-ended statements in response to the request for factors underlying the intelligibility of sung text. Many of the statements included references to more than one important factor. Each of these references was considered as a separate sub-statement, so the total number of sub-statements analyzed came to 851. These 851 sub-statements were considered independently by the two authors. Of these 851 sub-statements, 287 (34%) referred to the performer, 244 (29%) to the environment, 203 (24%) to the listener and just 117 (14%) to music and word-setting. Following discussion over any categorization differences, we extracted 43 separate factors categorized under the four headings suggested in the questionnaire; those placed by respondents under the “other” heading were re-categorized appropriately.

More than a third of sub-statements referred to the performer. The 15 performer-related factors, in other words those affecting intelligibility over which the performer has control, are listed in **Table 2** in order of the frequency with which they were mentioned.

**Table 2:** The number of statements (No.) and percentage (%) of performer-related factors affecting sung text intelligibility.

Factor	No.	%
Articulation, diction, enunciation	65	23
Balance between singer(s) and accompaniment	44	15
Communication of text, expression, and stage presence	29	10
Attitude, effort, preparation, projection	26	9
Volume	18	6
Voice quality and range	14	5
Consonants	13	5
Language/text: feel for/knowledge of/understanding	13	5
Technique and training	13	5
Choral ensemble	11	4
Pronunciation and accent	11	4
Vowels	10	3
Other	9	3
Performance style, vibrato	6	2
Breathing and phrasing	5	2

Total	287	100

Nearly a third of sub-statements referred to factors within the environment. The 10 environment-related factors, reflecting the listening situation or environment, and therefore not necessarily under either the performer’s or listener’s direct control, are listed in **Table 3**, above.

**Table 3:** The number of statements (No.) and percentage (%) of environment-related factors affecting sung text intelligibility.

Factor	No.	%
Acoustic	57	23
Location	50	20
Distraction	47	19
Amplification/reproduction	27	11
Proximity to performer	18	7
Visibility of singer(s)	17	7
Access to text	14	6
Situation	5	2
Use of electronic processing	5	2
Comfort	4	2

Total	244	100

Almost a quarter of sub-statements referred to the listener. The 11 listener-related factors, reflecting the listener’s psychological state and experience, are listed in **Table 4**.

**Table 4:** The number of statements (No.) and percentage (%) of listener-related factors affecting sung text intelligibility.

Factor	No.	%
Hearing ability	39	19
Attention	38	19
Familiarity with (knowledge of) repertoire (genre, specific work)	24	12
Motivation	23	11
Familiarity with language/accent	18	9
Desire to understand words	15	7
Familiarity with specific text	15	7
Mood, preference, receptiveness	14	7
Individual differences (listener’s age, sex, and health/fitness)	7	3
Background/experience	6	3
Values	4	2

Total	203	100

The remaining 14% of sub-statements referred to factors relating to the music and the word-setting, rather than those within the control of either the performers or the listener. They are listed in **Table 5**.

**Table 5:** The number of statements (No.) and percentage (%) of music- and words-related factors affecting sung text intelligibility.

Factor	No.	%
Genre	49	42
Relationship of music and words	25	21
Compositional style	15	13
Language	11	9
Tessitura, writing for voice	10	9
Words	4	3
Speed	3	3

Total	117	100

As explained in the section on Analysis, the respondents were then split into subgroups on the basis of (1) level of musical expertise (there were 76 experts and 59 non-experts), (2) rating of perceived understanding of sung text in both familiar and unfamiliar languages (108 respondents in all), (3) proportion of vocal and/or choral music listened to (67 devoted at least 75% of their music-listening time to vocal and/or choral music while 65 devoted less of their listening time to it), and (4) whether or not respondents reported being or having been singing teachers (a total of 18 as opposed to 117 who were not singing teachers). Note that not all respondents answered all questions.

Non-experts listed significantly more factors overall (*M* = 7.4, SD = 5.67) than experts (*M* = 5.4, SD = 4.81, *t*_133_ = 2.167, *p* = 0.032) and significantly more listener-related factors (non-experts *M* = 2.0, SD = 1.75; experts *M* = 1.2, SD = 1.40, *t*_133_ = 2.926, *p* = 0.004). An analysis of how expertise affected perceived importance of understanding sung text was then carried out. As above, we coded importance ratings on a scale of 1–3, with 1 = not important and 3 = very important. Of those who rated importance, experts (*n* = 60, *M* = 2.67) rated the understanding of texts in familiar languages significantly more important than did non-experts (*n* = 48, *M* = 2.40, *t*_106_ = 2.311, *p* = 0.023). This was also the case for unfamiliar languages, experts (*n* = 60, *M* = 1.98) rating the understanding of sung texts significantly more important than did non-experts (*n* = 47, *M* = 1.68, *t*_105_ = 2.316, *p* = 0.022).

A correlational analysis was undertaken to find out the nature of the association between rated importance of understanding sung text and number of factors listed. There were significant correlations between rated importance of understanding sung text, but only in a familiar language, and both the number of performance-related and environment-related factors listed (both *r_S_* = 0.208, *p* = 0.031). Thus the more important the understanding of sung text in a familiar language was deemed to be, the more factors were listed relating to the performer (e.g., diction) and the environment (e.g., room acoustic).

There were no significant differences between the numbers of factors listed, overall or within any specific category, attributable to whether more or less than 75% of respondents’ music-listening time was to vocal and/or choral music. Nor were there any significant differences between the numbers of factors listed by teachers and non-teachers. This was the case whether all participants were considered, or only those labeling themselves as experts.

## DISCUSSION

The survey proved to be a rich source of data, both qualitative and quantitative. The respondents, 18 of whom were singing teachers, had a broad range of singing expertise and were therefore broadly representative of vocal performers generally; they were also listeners to vocal and choral music, with half the respondents devoting at least 75% of their listening time to vocal and choral music. The main findings of the study were that it is important for listeners to understand sung text. They are most likely to attribute the extent to which it is intelligible to performers themselves, then the environment, then the listener and finally the words and music, including their relationship to each other. An unexpected finding was that respondents classified as “non-expert” proposed more potential factors underlying intelligibility than did expert musicians.

### IMPORTANCE OF UNDERSTANDING SUNG TEXT

It was necessary to ask the respondents how important it was for them to be able to understand sung words. If few respondents worried about being able to understand the words, then they would be unlikely to consider many factors affecting the understanding of sung text, and this could negatively affect the validity of the study. Overall, respondents’ ratings indicated that they believed it more important to understand sung text in a familiar language than in an unfamiliar language. This is not surprising, if the word “understanding” is taken to mean “comprehending” rather than merely “hearing clearly,” since words in an unfamiliar language convey no semantic meaning. As one respondent said,

“…understanding meaning” is so different from “picking out” with reference to whether it’s a language one knows or one that is less familiar. Have you considered age? I remember when studying nineteenth century German lieder and French chansons in my twenties, I was bowled over by the music and the gist of what it was portraying, but not understanding the individual meaning of words. It was all very romantic and I projected my own meanings onto the sung words (F, 56, music teacher, and amateur singer).

Intelligibility ratings for familiar and unfamiliar languages were strongly correlated. Respondents who ascribed greater importance to intelligible singing did so irrespective of whether the language was familiar to them. Thus, in the words of the respondent above, the importance of “understanding meaning” and “picking out” tended to match: either both were important, or neither. Sixty-one per cent of respondents rated being able to understand the text in a familiar language as very important; only 7% said it was not important. This could be related to genre – for instance, to follow the plot of an opera it can be crucial to understand the words, but this is not necessarily the case for pop songs – or listening context:

When I listen to Jazz songs in the background, the text is not important and I am just sometimes happy to grasp a phrase. When I listen during driving a car on a motorway, I want to understand the text I think (M, 28, semi-professional singer).

The findings do suggest, however, that making themselves understood is an important goal for singers to strive for, as this is appreciated by listeners (Collister and Huron, 2008); also, the majority of respondents felt that the understanding of sung text was sufficiently important for them to take the task of considering relevant factors seriously. Even in relation to listening to texts in an unfamiliar language, as large a proportion as 17% of respondents rated understanding the words as very important, with a further 51% rating it as quite important. Like “understanding,” “[un]familiarity” can be interpreted in different ways: listeners to texts in languages other than their own may not comprehend every word but still, if they can make them out, recognize enough to grasp their overall mood and meaning (in the context of Italian opera, for example, key words might be *amore* and *Dio*). Even without understanding the sense of individual words, listeners may deem a performance more enjoyable, complete or accomplished if they can hear them clearly, since this enables emotions to be communicated, and therefore the feeling of a connection between singer and listener. Experts (professional musicians, semi-professional singers, and student singers) rated the understanding of sung text significantly more important than non-experts, perhaps because experts rely on singing for part if not all of their income, and know that if they do not communicate the text clearly, in some genres at least, this could reduce the likelihood of their securing future singing engagements. The significant correlation between ratings for the importance of understanding the text in familiar and unfamiliar languages suggests that being able to make out the words is generally more important to some people, and less to others, irrespective of familiarity with a specific language. This could be because people listen to vocal and choral music in different ways: actively, with full attention, and more passively, when, for example, it is simply providing background atmosphere.

### PERFORMER-RELATED FACTORS

The respondents were asked to provide statements referring to factors relating to sung text intelligibility in four categories: performer-related, environment-related, listener-related, and music- and words-related. Overall, a third of the statements provided by respondents were related to the performer (Scotto Di Carlo, 2007a,b). Moreover, those respondents who rated understanding text in a familiar language as more important tended to provide more performer-related factors than those who did not. Most respondents were themselves performers, so they would have been aware of the impact that they as performers could have on listeners’ understanding. As discussed above, if respondents were not very interested in being able to understand the text, we would perhaps expect fewer performer-related factors to occur to them. Almost 60% of statements in the performer-related category included the four factors mentioned most often: diction and articulation (which was one of four example factors provided on the questionnaire); balance between singer(s) and accompanying instruments; communication and stage presence; and attitude, preparation, and projection (e.g., singer’s formant: Smith and Scott, 1980; Sundberg and Romedahl, 2009). With the exception perhaps of balance, these factors are all clearly under the control of the performer and, therefore, can be addressed by vocal pedagogy (Falkner, 1983; Adams, 1999; Emmons and Chase, 2006). They also concern communication with the audience, of both the semantic and the emotional meaning of what is being sung. Some respondents gave many such factors in their statements relating to the performer, for example:

Diction is essential – especially consonants. Why are Scottish singers better at this? Timing of choral diction all together now! Correct pronunciation in an agreed way. Practicing crispness by constant reminders and exercises like the tip of the tongue and the lips and the teeth! (F, 45, music teacher, and conductor)

and

Diction of words, and choice of diction depending on performance space; non-musical communication of meaning of words – through facial expression, body language, and movement; musical communication of meaning of words – emotional content (M, 27, PhD composition student, amateur/student singer).

Four per cent of performer-related statements, including the reference to “choral diction” in the first quotation, concerned choral ensemble, a topic explored in greater depth by Fine et al. (2008). Diction in choirs is clearly of paramount importance; it has to be managed carefully, since the more singers there are, the greater the acoustic variability.

Balance was often mentioned as affecting the intelligibility of sung text. This was usually related to the balance between the singer(s) and any accompaniment, whether piano, band, or orchestra (Scotto Di Carlo, 2007a; Sato and Prodi, 2009). Although listed as a performer-related factor, the performer is not the only contributor to balance; also relevant are the forces required by the composer, how loudly the instrumentalists play (or are directed to play by the conductor) and the use of amplification:

… the balance between the singer and accompanying instruments can affect intelligibility, as too loud an accompaniment can swallow the words and certain sounds and timbres can make picking out the words harder (F, 20, music student, and occasional singer).

Balance does not have to be equal: one respondent commented “if the singer dominates I find this can be as distracting as the accompanist dominating” (M, 44, lighting designer for opera company), although it must be acknowledged that finding something “distracting” does not necessarily mean the words are harder to make out.

Communication and stage presence were likewise considered to be important contributors to sung text intelligibility (Dunn, 1997). Communication and stage presence would be expected to contribute to both the overall performance quality, and the listener’s general understanding of the piece. Perhaps they also provide context so that potentially ambiguous words can be understood. For instance, respondents felt that “emotional expression and communication of the narrative/meaning” (F, 38, lecturer, and amateur singer) and “non-musical communication of meaning of words – through facial expression, body language, and movement” (M, 27, PhD composition student, and occasional singer) enhanced the intelligibility of sung text. As discussed above, in the context of the importance of understanding, one respondent thought this was true even for unfamiliar languages: “Emotional engagement – the message of the text can be conveyed even if the language is unknown to the listener” (F, 28, semi-professional student singer), although this respondent may be talking more about communication of general meaning than about understanding the actual words.

It was noticeable that several respondents mentioned vocal range, vowels, and consonants. Sundberg and others (see Sundberg, 1987, 2012 for reviews) have shown many times that sopranos are harder to understand than lower voices, as the formant frequencies of the vowels exceed the resonant frequencies of the vocal tract. Our respondents are clearly aware of this, one commenting “Certain types of singer are harder to hear than others. Sopranos modify their vowels often…,” although they did go on to say “…and basses are frequently just hard to hear at all” (M, 39, semi-professional singer, and musicologist). The point about basses is no doubt that low notes in the male register tend to lack power and do not carry well, also affecting intelligibility. However, syllables are easier to understand than isolated vowels, perhaps due to the presence of acoustic transitions (Smith and Scott, 1980), and although vowel modification is likely to affect intelligibility, at least in sopranos, the presence of consonants and other contextual phonemes can no doubt assist understanding. One way that singers can aim to improve intelligibility is to modify vowels and consonants (Dunn, 1997; Hollien et al., 2000; Ginsborg, 2014). According to one respondent:

To sing intelligibly the singer must inevitably distort or elongate certain spoken vowel sounds in order to discover the true sung vowel sound allowing a note to be sustained etc. (M, 41, professional singer),

although another respondent felt this did not necessarily assist intelligibility:

In classical singing, the pronunciation (especially vowel sounds) is often made subservient to musical quality, resulting in significant distortion of the words (M, 51, amateur singer).

One respondent argued that this related to the singer’s own familiarity with the language: “some singers distort sounds in languages other than own” (F, 59, psychologist, and amateur singer). Another suggested

… especially if you are only fairly familiar with the language – facial expression is crucial. Expressiveness makes for a much more meaningful performance (F, 49, dyslexia specialist, and semi-professional singer),

but whether she was talking about how a singer can enhance general communication of emotion and meaning, or the intelligibility of the words *per se* is unclear from her quotation.

Other performer-related factors mentioned by the respondents pertained to training and experience, including attitude, preparation, feel for/knowledge of/understanding of the language, or specific text, and technique and training. Together, these suggest that many respondents felt that sung text intelligibility is clearly under the control of the singer, and that preparation, whether formal training or informal familiarity with the text, can help to improve sung text intelligibility.

### ENVIRONMENT-RELATED FACTORS

The second most popular category of factors was that which related to the environment. Those respondents who rated understanding sung text in a familiar language as more important tended to suggest a greater number of environment-related factors than those who did not. As discussed above, this is probably because most respondents were performers, and therefore aware of the ways in which the environment can affect singers’ ability not only to communicate, generally, but also enhance the intelligibility of sung text. Almost 75% of statements in the environment-related category included the four factors mentioned most often: acoustic, location (the example provided on the questionnaire), distraction, and amplification. Apart from distraction they all relate directly to the concert situation (distraction may or may not do so), and many respondents specifically identified echoes and reverberations, which are well known to have a detrimental effect on the intelligibility of text both spoken and sung (Bradley et al., 1999; Boothroyd, 2004; Scotto Di Carlo, 2007a; Yang and Bradley, 2009; Rychtáriková et al., 2011). As one respondent asked, “Have you ever tried listening to music in York Minster^1^?” (M, 34, teacher, and amateur singer). A second commented on the effects of concert location, nature of amplification system where used and building design on resonance and acoustic, while a third focused on the implications of the environment, more generally, for the singer:

Yes, always more difficult outdoors if not enhanced. If enhanced, the quality of the system is paramount. Internal size of building matters + resonance of building/seating, number of people in audience, shape of building, e.g., non-parallel walls, etc. (F, 62, doctor, and amateur/student singer).

Depending on the environment, I may have to enunciate more, and that may help in understanding the meaning of the text and getting across the meaning to the listener, heightening expressivity of the music/text (M, 24, student singer).

Distraction may be listener-related in that different people have different capacities for attention – some are more distractible than others – and are therefore capable of carrying out a range of other activities while listening to music. It is also environment-related because the listeners’ activities and a variety of other extra-musical stimuli can divert their attention from the words being sung, even if they are trying to pay attention to them. Optimal listening conditions were identified as follows:

No or very little background noise. Indoors probably easier. Good headphones (brings the sound much more sharply within the ear’s “focus”). Good stereo system/speakers (M, 28, semi-professional student singer).

Moreover, distraction does not have to be auditory to impair sung text comprehension: other responses referred to scenery, people, architecture, and other visual distractions, such as those experienced by audiences for *son et lumière* performances.

Several respondents mentioned amplification as being important in determining the intelligibility of sung text. Although little research has so far investigated the effect of amplification on understanding sung text (Doria et al., 2012), it was felt that both “the quality of the amplification” (M, 62, retired academic, non-singer) and the “quality of a recording (if it is appreciation of recording)” (F, 34, academic, and amateur singer) could affect the ease with which listeners could understand sung words. It was also pointed out that the use of amplification relates both to genre, and to location: “if outdoors, appropriate amplification and a good microphone. Singer must use microphone well, i.e., not too close if volume is loud etc.” (F, 39, teacher, and amateur singer). Use of microphones is undoubtedly genre-specific, and it is the case that poor technique (for instance, holding the microphone too close to the mouth) can impair rather than enhance the listener’s understanding of the words being sung.

Other environment-related factors mentioned by several respondents included their proximity to and (in some cases, therefore) the visibility of the singer. It is usually easier to understand speech when the listener can see the speaker’s lips (Sumby and Pollack, 1954; Samuel, 2011), and some respondents reported this to be the case for sung text (Jesse and Massaro, 2010): “Proximity to source of music/volume. Ability to see the performer: a certain amount can be deduced by lip reading and from the actions of the performer” (F, 56, amateur singer). The listener’s comfort was also mentioned, one respondent noting that a “terribly cold cathedral will always have some bad impact on appreciation of choral performance” (M, 34, amateur singer, and musicologist), and another remarking that an environment that was too hot could lead to “soporific inattention” (M, 62, medical practitioner, and amateur singer). Again, however, it is unclear whether “an appreciation of choral performance” refers specifically to understanding the words, though the latter is undoubtedly a component of this appreciation.

### LISTENER-RELATED FACTORS

A quarter of the statements were related to the listener. Seventy per cent of statements in the listener-related category included the four factors mentioned most often: hearing ability (the example provided on the questionnaire), attention, familiarity, and motivation. The ability to hear is primarily physiological, and therefore likely to decrease in older age, impairing the understanding of speech (Humes, 1996), and presumably also sung text. Not only can background noise exacerbate pre-existing hearing problems (Lorenzi et al., 2006), but, as already discussed, it can be hard to attend to one stimulus in the presence of others and sung text is much more likely than speech to be accompanied by other sounds. Like attention, familiarity and motivation are cognitive factors. Listeners are more likely to be able to understand the words if they pay attention to the performer(s), and this will probably be enhanced if they are motivated to listen to the words. Attention should be distinguished from distraction, as – depending on their attentional capacity – listeners can control their levels of attention, whereas distraction usually results from external stimuli, beyond the listener’s control. One respondent reported his listening strategy as follows: “Engaging with the singer(s) and actively listening rather than letting the music ‘wash’ over” (M, 41, professional singer). Motivation, or a desire to understand the words, can clearly influence one’s ability to understand: “There has to be a desire to make the effort to understand, as this is seldom effortless” (F, 52, amateur singer), and this may also relate to the genre of the music and any additional activity being undertaken by the listener:

… the listener’s attention, whether s/he focuses his or her attention on the text or on one or more musical instruments; whether the listener concentrates on the act of listening or has put on some music as a backdrop to other activities (e.g., housework) → distraction (F, 26, professional singer).

Research on repetition priming has shown that people are more efficient at processing stimuli with which they have already been presented than novel stimuli (e.g., Forster and Davis, 1984; Orfanidou et al., 2006). “Processing” includes recognizing, categorizing, and identifying, so it is not surprising that respondents reported finding it easier to attend to and understand sung text when they were familiar with the work, the musical genre or even the language being sung, singer’s accent, and the specific text, although these were mentioned less often. Gass and Varonis (1984) found that familiarity enhances the comprehensibility (i.e., subjective ease of understanding) of non-native speech; similarly, one respondent observed that “Familiarity with the source text, e.g., standard liturgical texts, poems etc. [allows people to] ‘fill in’ any parts which can’t easily be picked up by listening” (M, 37, amateur singer), as does “Having a copy of the words of unfamiliar pieces” (M, 59, amateur singer). Given that many respondents were singers and singing teachers, it may well be that the importance they attributed to familiarity with the repertoire arose from their own experiences as performers.

### WORDS AND MUSIC-RELATED FACTORS

The remaining 14% of statements related to the piece itself, both in terms of its words and their setting to music. Seventy-five per cent of the statements in this category included the four factors mentioned most frequently: genre (the example provided on the questionnaire); relationship between the words and the music; compositional style; and language. Respondents perceived sung words to be easier to understand in some genres (e.g., jazz) than others (e.g., opera), as demonstrated empirically by Condit-Schultz and Huron (2013). Singers are trained to fulfill the requirements of different genres. For example, because pop singers are amplified they do not use the classically trained singer’s formant, required particularly by operatic singers to project their voices above the orchestra, and can produce smaller deviations from spoken vowels (Sundberg, 2012); performers in musical theater often employ the “belt” technique (DeLeo LeBorgne et al., 2010) involving enhanced vibrato and “ring” to increase loudness. Some genres require additional strategies, as one respondent explained:

Genre of music is very important; in some genres, such as opera, the words can often be very hard to understand, whereas madrigals etc. are usually very clear. In more popular genres this becomes even more important because of the wide range of vocal styles employed, which often include extreme techniques such as “screaming” or “growling” the lyrics, often making them hard to understand (F, 20, occasional singer).

Listeners do not always feel it important to understand the words, although the role of the performer in deciding the extent to which this is considered a priority is nevertheless acknowledged:

Obviously some genres are impossible to understand what they are singing for example heavy rock but it depends on the individual and how much importance they place on diction (F, 20, student singer).

References to the relationship between the words and music concerned the use of melisma and, especially in polytextual music, part-writing. These issues arise not only from compositional style but also composers’ motivations and decisions, whether or not they are conscious of them at the time of writing:

Relationship of words to music, e.g., text-setting; relationship of singer to other forces; importance of words to the composer; intelligibility of words as a poem/prose (F, 20, student singer).

Composers who choose to set texts that are relatively well known, in the context of a liturgical mass, for example, or that are repeated many times, as in some operatic arias, may think of the voice primarily as an instrument, focusing to a lesser extent on the communication of semantic meaning; in both cases intelligibility may be reduced. Similarly, it can be hard to understand poems that might be considered “dense” in that they contain little redundancy [e.g., Dylan Thomas’ *The Force That Through the Green Fuse Drives the Flower*, set to music by Huub Kerstens in 1984] although sung ensembles involving the simultaneous performance of different texts may be intelligible by virtue of their having already been presented individually to the audience: examples include the sextet “Riconosci in questo amplesso” from Act 3 of Mozart’s *Le Nozze di Figaro,* the quartet “Bella figlia dell’amore” from Act 3 of Verdi’s *Rigoletto*, and the ensemble “Tonight” from Bernstein’s *West Side Story*. Commenting on the effect of compositional style on intelligibility, one respondent wrote

Homophonic music is clearer than polyphonic. Early Renaissance music sometimes has 4 texts simultaneously! Big choral works have the danger of being drowned by the instruments. Chamber music or unaccompanied works are clearest (F, 45, semi-professional singer, music teacher, conductor),

and another, reflecting on his own experience as a performer, said

Finding the balance between the musical interpretation of a work and the meaning of the words can at times be tricky. It is helped when songs, arias etc. have been composed well and the words set well. This, in my experience makes performing them much easier. Composers who have not written for singers before I know can find it difficult and I have enjoyed working with several composers exploring what they and I can and can’t do. As a singer I believe that clear diction and a considered and thoughtful delivery of the text whilst observing the musicality of a piece is essential (M, 30, professional singer).

Some respondents felt that it is always important for listeners to have access to the text irrespective of their (assumed) familiarity with its language. Both authors sing with choirs whose printed programs are more likely to include the texts of works in foreign languages than those in English, but some respondents felt that the decision as to whether the text should be provided must depend to some extent on genre:

Items regardless of language should always either have the words printed in a program or use surtitles unless the setting is extremely informal, e.g., a folk music concert (F, 25, student singer).

Surtitles or subtitles, often used in opera houses, are particularly welcomed by members of the audience for whom the language of the libretto is unfamiliar. They are also welcomed, however, by “native” audiences. With very few exceptions, English National Opera gives all its performances in English or English translations; since 2005 English subtitles have been provided, “making [opera in English] accessible to English-speaking audiences,” with certain seats having “a good view of the subtitles, so no twist or turn of the plot goes unnoticed” (Enjoy Opera, 2014).

### EFFECTS OF OTHER DEMOGRAPHIC VARIABLES

Overall, one unexpected finding was that the statements of those respondents subsequently categorized as non-experts (amateur singers and non-singers) yielded significantly more factors than experts, both overall and for listener-related factors. One possible explanation is that non-experts did not rule out certain factors believed by experts not to affect sung text intelligibility. If experts had listed significantly more performer-related factors than did non-experts, it might have been concluded that experts were drawing on their own experience when completing the questionnaire, leading them to focus more on performer-related factors than those in other categories, particularly listener-related factors. Whatever the explanation, it cannot be disputed that the statements made by the whole sample of respondents yielded a very broad list of factors overall.

Two variables that were not found to affect the factors listed by the respondents were the proportion of music listening time devoted to listening to vocal/choral music, and whether or not the respondents were singing teachers. Although half the respondents listened to vocal/choral music for more than 75% of their music listening time, this statistic was negatively skewed, with a mean of only 65%, with over half (53%) of respondents stating that the percentage of music they listened to defined as vocal or choral music was 50%, 80%, or 90%. Singing teachers might have been expected to list more factors than non-teachers, or at least performer-related factors, as these are potentially under their control through their teaching practice. No difference was found between the two groups, however, perhaps because the sizes of the two groups were unequal: only 15% of the sample were singing teachers.

### LIMITATIONS AND FUTURE RESEARCH

While it should be remembered that the study was designed to be exploratory in nature it is nevertheless worth addressing its limitations. The sample was biased toward respondents from Western first-world countries (a quarter were from 11 countries outside the UK but none was from Asia or Africa) and musicians with Western classical training. It is possible that respondents from other musical cultures would have different attitudes toward the importance of understanding sung text, suggest factors not listed by the respondents to the present survey, and prioritize different factors. It would be interesting to repeat the survey with respondents from a greater variety of countries and cultures, including performers of and listeners to different types of world music. The present survey was aimed at respondents thought likely to have experiences analogous to those of the authors: the majority were practicing musicians, at least half of whom were expert singers. Performing musicians represent a very small minority of the wider population, however, so survey responses from those who listen to vocal and/or choral music but are not themselves singers or instrumental musicians could be informative. Although it is unlikely that new factors would be identified, the relative importance of existing factors might well be different for such a sample. It could also be argued that as many participants were trained singers, and thus likely to be have been drilled in the importance of diction, there is a danger of a self-fulfilling prophecy, in that we would expect such participants to believe it very important that sung words can be understood. However, the point of including trained singers in the sample and asking participants how important they thought it was for singing to be intelligible was to verify that they had an interest in the issue and would be likely to suggest many factors. Investigating how training affects both the importance to the individual of understanding sung text and the factors perceived to affect sung text intelligibility was not our aim in the present study.

Factors could have been categorized in different ways, requiring the authors to interpret respondents’ statements with caution and make decisions with which others might disagree. For instance, “balance” was clearly important to many respondents. The authors agreed to categorize this as a performer-related factor, on the grounds that singers are capable of producing louder and more focused sounds themselves, thus controlling dynamics and projection. Yet the environment could play an equally important role: a resonant acoustic boosts certain frequencies over others, accompanying instrumentalists and/or conductor influence the volume of sound produced by the soloist or ensemble of singers; and the composer can demand larger or smaller accompanying forces, as in the case of the solo soprano accompanied by an orchestra of Wagnerian proportions or a single instrument, typically a piano. One respondent commented “I found it difficult to know where to list certain points in answer to the previous question, e.g., is the use of a microphone concerned with environment, musical genre, performer or all three?” (M, 62, retired academic, and non-singer). Similarly, the two researchers’ initial categorization of statements into specific factors was not always identical, necessitating discussion. This uncertainty over categorization, either into factors or categories of factors, does not, however, invalidate the study, but rather emphasizes the interconnectedness of the myriad aspects that can affect sung text intelligibility, illustrating its complexity.

In order to clarify what was meant by the four category headings of performer, listener, environment, and words and music, exemplars were provided on the questionnaire for each category. This produced a possible bias insofar as the exemplars were mentioned most or second most often in their category, perhaps over-inflating their perceived importance for sung text intelligibility. The questionnaire suggested only four factors, however, while respondents mentioned a total of 43: almost ten times as many new factors as those provided. Their use was felt, therefore, not to decrease the validity of the results.

As the data collection was carried out through a mainly internet-based survey, with a small proportion of completed paper questionnaires, there was no monitoring of the respondents’ qualitative responses via interviews, for example. Although it is likely that being able to make out and understand the words can contribute to the overall perceived quality and enjoyment of the musical performance, it is also possible that some respondents lost sight of what they were being asked, instead listing factors that they thought enhanced the performance, ignoring the specific issue of sung text intelligibility. Two examples, both given above, were when a singer dominating an accompanist can be “distracting,” and the fact that expressiveness can create a more “meaningful” performance. Although both these points are pertinent to the overall quality of performance, they do not necessarily relate directly to intelligibility. Overall, however, this also emphasizes the complex relationship between intelligibility and other facets of performance quality.

Finally, it should be noted that this exploratory study identifies only those factors that respondents believe are important. This is an important first step toward illuminating the issues that might indeed affect sung text intelligibility in practice, and suggesting specific factors that can be investigated in an objective, empirical fashion. Some of the points made in the introduction involve assumptions about the similarity between speech and sung text, and thus extrapolate from empirical evidence concerning speech perception. Many of these assumptions, as backed up by our participants’ views, can and should be empirically tested (as has been done, for instance, by Collister and Huron, 2008). Follow-up research by the present authors is already under way, and is investigating the impact of the number of singers and the meaningfulness of the text on intelligibility, for example (Ginsborg et al., 2011), but a detailed discussion of the findings is outside the remit of this article. “Number of singers” relates to the distinction between solo and choral singing, mentioned by several respondents. “Meaningfulness of the text” relates to the finding that spliced spoken words are harder to identify than in context (Lieberman, 1963) and the respondent’s comment (shown above) regarding the importance of intelligibility when texts such as poems are spoken aloud. This research and further empirical studies manipulating variables identified in the present study will enable us both to validate the views of musicians presented here, and to increase our understanding of practical ways of enhancing the intelligibility of sung text, such as improving singers’ diction through more effective vocal pedagogy.

## Conflict of Interest Statement

The authors declare that the research was conducted in the absence of any commercial or financial relationships that could be construed as a potential conflict of interest.

## Footnotes

1 An English cathedral with a reverberation time at 1 kHz of 7.65 s (Murphy and Shelley, 2010; Shelley and Murphy, 2014).

## Appendix a – Questionnaire

1. Sex (male/female):
2. Age (years):
3. Nationality:
4. Profession:
5. Singing experience (please circle the appropriate response)
a. occasional singer, for pleasure or work
b. amateur singer
c. student singer
d. semi-professional singer
e. professional singer
f. other (please specify)
6. Are you a singing teacher (please circle the appropriate response)?             Y           N
7. If so, how many years’ experience of teaching singing do you have?
8. What genre(s) of music do you listen to most often? Please list them below.
9. What percentage of the time that you spend listening to music do you listen to vocal and/or choral music, as opposed to purely instrumental music?

There are potentially many factors that contribute to listeners’ enjoyment of vocal and/or choral music. The one that we are exploring in this study is intelligibility: being able to understand the meaning of the words, if the text is in a language with which the listener is familiar, or being able to make out the words in an unfamiliar language.

10. How important is intelligibility to you (please circle the appropriate responses)?
a. *Familiar language*:
Not at all important Quite important Very important
b. *Unfamiliar language*:
Not at all important Quite important Very important

There are potentially many factors affecting the intelligibility of sung text. These could pertain, for example, to the listener, the environment, the music and the performer. Please provide a list of the factors that you consider important for the intelligibility of sung text under the following headings:

11. Listener (e.g., hearing ability)
12. Environment (e.g., location: indoors/outdoors)
13. Music (e.g., genre)
14. Performer(s) (e.g., diction)
15. Other

Please make any further comments about this questionnaire or the research in general, if you wish, below:
